# Is Sleep Duration Associated with Biological Age (BA)?: Analysis of (2010–2015) South Korean NHANES Dataset South Korea

**DOI:** 10.3390/ijerph15092009

**Published:** 2018-09-14

**Authors:** Kyu-Tae Han, Dong Wook Kim, Sun Jung Kim

**Affiliations:** 1 Research and Analysis Team, National Health Insurance Service Ilsan Hospital, Goyang 10444, Korea; kthan.phd@gmail.com (K.-T.H.); kimdw2269@gmail.com (D.W.K.); 2 Institute of Health Services Research, Yonsei University College of Medicine, Seoul 03722, Korea; 3 Department of Health Administration and Management, Soonchunhyang University, Asan 31538, Korea

**Keywords:** biological age, sleep duration, self-management, metabolism

## Abstract

(1) Background: South Korea ranked worst in sleep duration compared to other countries, but there are no clear healthcare programs to guarantee sufficient sleep. Studies are needed to suggest evidence and arouse public awareness of the negative effects of abnormal sleep duration. In this study, we investigated the relationship between biological age (BA) and sleep duration. (2) Methods: We used data from the Korea National Health and Nutrition Examination Surveys (KNHANES V-VI; 2010–2015, which is an annually cross-sectional study including 29,309 participants). We performed multiple linear regression to investigate the associations between sleep duration and differences in BA and chronological age (CA). (3) Results: A total of 14.22% of respondents had short sleep duration (less than 6 h per day) and 7.10% of respondents had long sleep duration (more than 8 h per day). People with long sleep duration had a positive correlation with difference between BA and CA (>8 h per day, β = 1.308, *p*-value = 0.0001; ref = 6~8 h per day, normal). Short sleep duration had an inverse trend with the difference, although the result was not statically significant. Associations were greater in vulnerable populations, such as low income, obese, or people with chronic diseases. (4) Conclusions: Excess sleep duration that is greater than the normal range was associated with increased BA. In particular, such relationships that are related to worsening BA were greater in patients with low income, obesity, and chronic diseases. Based on our findings, healthcare professionals should also consider the negative effects of excess sleep, not only insufficient sleep. Alternatives for controlling optimal sleep duration should be reviewed, especially with vulnerable populations.

## 1. Introduction

Sleep plays a major role in daily life by assisting physical and mental recovery. Deficiencies in sleep quality or duration can contribute to several health problems, including heart disease, hypertension, diabetes mellitus, and stroke [1,2,3,4,5]. Sleep is an essential factor for health, and its importance is now well-established. South Korea has experienced rapid social and economic developments since the late 20th century, and most people now have busy daily lives [6]. As a result, South Koreans have sleep difficulties. The Organization for Economic Cooperation and Development (OECD) reported that South Korea ranked in the worst level for sleep duration when compared to other countries (South Korea: 469 min per day, 18th/18; United States: 518 min, 2nd/18; UK: 503 min, 11th/18; and, OECD average: 502 min per day) [7].

Some programs and policies have been introduced to improve the quality and quantity of sleep. Nevertheless, problems that are related to sleep remain. Patients diagnosed with sleep disorders have exponentially increased, according to a report of the Health Insurance Review and Assessment Service (HIRA); 287,835 patients had sleep disorders in 2010, which increased to 494,915 patients with sleep disorders in 2016 [8]. Reflecting this situation, nap cafés that operate during lunchtime and target office workers have become popular in South Korea [9]. However, some studies suggest that excess sleep duration can also cause negative health outcomes. Optimal sleep duration, rather than merely increased sleep duration, is suggested as a key factor in managing sleep health [10,11].

To solve the emerging health problems that are related to sleep, increased public attention and healthcare resources are key factors. Thus far, few alternatives have been introduced due to the social atmosphere and limited awareness of sleep problems. In addition, other than programs for management of sleep apnea, there are no clear healthcare programs to promote sufficient sleep duration [12]. Thus, evidence is needed to arouse public awareness of the negative effects of abnormal sleep duration. Further information would be helpful for establishing optimal alternatives to abnormal sleep duration in South Korea.

In this study, we will investigate the relationship of sleep duration and biological age (BA). With aging, chronological age (CA) could no longer reflect physiological function, general health, or overall decline, because an individual’s health status depends on different self-management behaviors and characteristics [13,14]. Thus, an index that can substitute CA to evaluate whether actual health status is needed. BA, which is estimated by measuring health status biomarkers, has been used to estimate physiological function, overall health status, and aging. BA is a useful index that enables subjects to understand their health status and emphasizes the importance of a healthy lifestyle; it has attracted increasing attention since 2000, and many factors, such as socioeconomic status, nutrition, physical activity, and biochemical/hormonal mechanism that affect BA are now known [15,16,17,18]. However, sleep is a major part of daily life that was not assessed as a factor that could affect BA. To investigate the relationship between sleep duration and BA, we study the negative effects of abnormal sleep duration, including worsening BA and cardiovascular diseases. 

## 2. Materials and Methods 

### 2.1. Study Population

We used data from two Korea National Health and Nutrition Examination Surveys (KNHANES V and VI; 2010–2015). The KNHANES is a cross-sectional study conducted annually by the Korean Centers for Disease Control (KCDC) using a stratified, multistage, cluster sampling design. The surveys include three questionnaires: Health Interview Survey, Health Examination, and Nutrition Survey. All of the participants were interviewed by trained personnel during the health examination. In this study, respondents who did not provide data that would have enabled BA calculation and those <20 years of age were excluded, as were subjects who did not report their sleep duration. We ultimately included 29,309 eligible participants.

### 2.2. Variables 

The BA that was used in this study was calculated with object for estimating risk of metabolism. First, correlation analysis between CA and metabolism diagnostic parameters, including waist circumstance, systolic blood pressure, diastolic blood pressure, fasting blood sugar level, triglycerides, and high-density lipoprotein cholesterol was performed. Systolic blood pressure and diastolic blood pressure were converted into mean arterial blood pressure for the exclusion of redundancy and application of both parameters. After then, principal component analysis (PCA) was conducted for age and the five metabolism diagnostic parameters. The factor with the highest eigenvalue was decided as the principle component, and BA score was calculated using the first principal component obtained. Regression analysis was performed while using the PCA variables as dependent variables and the five metabolism biomarkers as independent variables. The formula developed after this process for estimation of BA by sex is as follows [19,20,21]:

*BA in males* = −76.0965 + 0.541 × (*waist circumstance*) + 0.271 × (*mean blood pressure*) + 0.213 × (*fasting blood glucose level*) + 0.059 × (*triglyceride level*) − 0.312 × (*high-density lipoprotein cholesterol level*) + 0.850 × (*age*) (1)

*BA in females* = −66.530 + 0.484 × (*waist circumstance*) + 0.328 × (*mean blood pressure*) + 0.303 × (*fasting blood glucose level*) + 0.080 × (*triglyceride level*) − 0.282 × (*high-density lipoproteins cholesterol level*) + 0.601 × (*age*) (2)

These parameters that are related to metabolism, including waist circumstance, mean blood pressure, fasting blood glucose level, triglyceride level, and high-density lipoprotein cholesterol level in calculating BA were measured through health examination on survey date. Then, we calculated the difference between BA and CA (BA-CA) to evaluate the actual health status of each individual with reference to BA. If the difference was positive when subtracting CA from BA, the individual was considered to have a worse health status when compared to similar age groups. Thus, the difference was considered an outcome variable in this study.

The primary variable of interest was sleep duration which collected based on question about “How many hours do you usually sleep?” in Health Interview Survey. It was categorized as follows: (1) less than 6 h per day (low), (2) 6~8 h per day (normal), or (3) more than 9 h per day (high). We also included other covariates in this study. Based on the previous studies, the risk of metabolism that related to BA in this study were differed by characteristics of study population, including general and socioeconomic status. Therefore, we included general characteristics, such as sex, age, educational level, marital status, economic activity, and household income for controlling the difference due to socioeconomic status [22]. Subjects were grouped by age as follows: <30 years, 30–39 years, 40–49 years, 50–59 years, and ≥60 years. Marital status was defined as follows: married, divorced/separated/bereaved, and single. Also, the prevalence of metabolism, including five parameters, were correlated with increasing BMI [23]. Subjects were grouped by BMI as follows: BMI < 23 kg/m^2^, underweight or normal; BMI = 23–25, overweight; and BMI > 25, obese [24,25]. By the aspects of healthy behaviors, physical activity was closely associated with metabolism, and the alcohol intake or smoking had positive association with increasing risk [26,27]. Aerobic exercise habits was defined as whether people take a moderate exercise more than 150 min a week or a high-strength exercise more than 75 min a week. High-risk drinking was defined as consumption of more than seven (males) or five (females) drinks on a single occasion at least twice a week. On the other perspectives, stress is also risk factors in chronic symptoms, including metabolism [28]. Stress awareness was defined as people who responded as “frequently” for the question about “How many perceived stress in daily life?” Hypertension, dyslipidemia, and diabetes mellitus were included due to close associations with diagnosis of metabolism [29].

### 2.3. Statistical Analysis

We first examined the frequencies and percentages of the study population to suggest the distribution of the study population by each categorical variable. Next, we performed t-tests and analysis of variance (ANOVA) to identify relationships between independent variables, BA, and BA difference (BA-CA). Finally, we performed multiple linear regression to investigate the association between sleep duration and difference (BA-CA) after adjusting for covariates. In addition, we performed subgroup analyses by household income, BMI, and diagnosis of chronic diseases (hypertension, dyslipidemia, or diabetes mellitus). We applied a sampling weight to each participant in order to generalize the data. SAS version 9.4 (Cary, NC) was used for all of the analyses. Data used in this study was collected after approving by the KCDC Institutional Review Board, and all of the participants provided written informed consent (2010-02CON-21-C, 2011-02CON-06-C, 2012-01-EXP-01-2C, 2013-07CON-03-4C, and 2014-12EXP-03-5C). In addition, all data was anonymized to avoid identifying the individual. 

## 3. Results

A total of 29,309 respondents participated in this study. Table 1 shows the distribution of the study population by each independent variable, including sleep duration. Among the study population, 14.22% of respondents had short sleep duration (less than 6 h per day) and 7.10% had long sleep duration (more than 8 h per day). 

Table 2 shows the mean and standard deviation of BA and difference (BA-CA) for each categorical variable. Overall, the average BA was 49.75 years and the difference was 4.00 years. People with short sleep duration had higher BA than other groups. People with long sleep duration had a higher BA-CA difference (*p*-value < 0.0001). Higher BMI was associated with higher BA and BA-CA difference (*p*-value < 0.0001). Health behavior indicators of aerobic exercise, smoking, and alcohol also generally had positive trends with higher BA and BA-CA difference. Diagnosis of a chronic disease, such as hypertension, dyslipidemia, or diabetes mellitus showed a similar trend to BA and BA-CA difference. 

Table 3 shows the results of linear regression analysis for BA-CA difference to investigate the association with sleep duration. People with long sleep duration had a positive correlation with the difference between BA and CA (>8 h per day, β = 1.308, *p*-value = 0.0001; ref = 6~8 h per day, normal). Short sleep duration had an inverse trend with BA-CA difference, although the results were not statically significant. Females or those in the 40~49 age group had higher BA-CA differences than other groups. On the aspects of health behavior, people with no aerobic exercise, smoking, and alcohol had higher BA-CA differences. Higher stress in daily life was associated with higher BA-CA differences. The diagnosis of chronic diseases, such as hypertension, dyslipidemia, or diabetes mellitus had a positive correlation with higher BA-CA difference.

**Figure 1:**
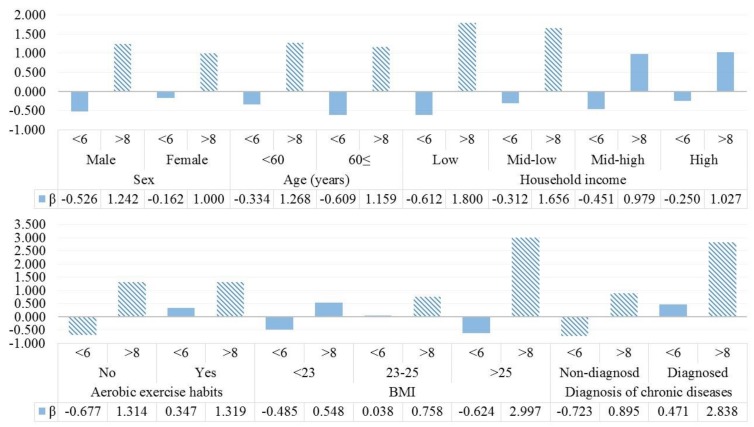
The results of sub-group analysis for the association between sleep duration and difference in biological age (BA) with chronological (CA) according to household income, BMI, and diagnosis of chronic diseases. Hatched bars show the statistically significant results.

We also performed sub-group analysis for linear regression analysis between sleep duration and BA-CA differences to investigate BA-CA differences, according to household income, BMI, and diagnosis of chronic diseases. The results are shown in Figure 1. Larger differences between BA and CA with longer sleep duration were more prevalent in lower income groups than others. However, there were no statistically significant results in the short sleep group (less than 6 h per day). In the results by BMI or diagnosis of chronic diseases, the increases in BA with long sleep duration were more frequent in obese groups or people with the diagnosis of chronic diseases. 

## 4. Discussion

Rapid socioeconomic development in South Korea has resulted in conflicting outcomes. Life is more convenient than in the past, but to survive under competition, most people experience worse mental health and stress than in the past. In particular, sleep duration is one major recent problem. Although sleep occupies about half of daily life, problems that are related to sleep have not been majorly addressed so far, and sleep alternatives are not sufficient [12]. The continuously poor management of sleep duration in South Korea must be addressed in near the future, and optimal management of sleep duration will play a major key role in sleep medicine [10]. This study focused on sleep duration and investigated the association between sleep duration and BA by considering the outcomes that can cause worsened sleep. 

Our findings suggest that sleep duration longer than the normal range could cause negative health outcomes. In particular, it was associated with higher BA than CA, similar to previous studies. Short sleep duration could contribute to negative health outcomes, such as obesity, stroke, and other cardiovascular diseases [1,2,3,4,5,11]. However, excessive sleep duration can also worsen individual health outcomes [30]. Related studies showed that excessive sleep duration was associated with depression [31]. In addition, some studies have suggested that excessive sleep is associated with dementia, nervous anxiety, and other chronic diseases [32]. Even worse, sleep duration longer than the normal range could shorten life expectancy [33]. Although some previous findings also suggested negative results with short or excess sleep duration, such studies commonly focused on health outcomes, such as incidence of chronic diseases. Thus, we analyzed the association between sleep based on optimal sleep duration (6~8 h) and BA. In addition, negative health outcomes due to excessive sleep were frequently caused due to loss of sleep quality in the previous findings [34]. However, its association between difference with BA and excessive sleep in this study can be explained as following mechanism. First, the physical activity was would be key role in managing metabolism, but excessive sleep could bring the loss of physical activity. Second, people with lethargy are more likely to vulnerable in controlling self-management, such as sleep duration. On the other hand, deficit of sleep was not associated with difference between CA and BA; it could support the results by excessive sleep. With the emerging importance of self-management, findings related to BA and sleep duration will be helpful in improving individual health. A nationwide health policy with limited resources must be efficiently managed to ensure the proper control of latent risk factors.

Sub-group analysis showed more instances of worsening BA with excess sleep duration in specific groups. Low-income groups were more affected by the relationship between sleep duration and BA, likely because worsening outcomes due to excess sleep duration have a greater impact on those with limited healthcare resources. Results suggest that the presence of other chronic diseases was highly associated with worsening BA and longer sleep duration. Therefore, excess sleep duration has more influence on increased BA in economically or clinically vulnerable populations. Healthcare professionals and policymakers should consider problems that are related to sleep duration and review possible solutions to the problem of sleep duration. Because potential risk factors have greater effects on vulnerable populations, efficient alternatives must be considered.

Our study has some strengths when compared to previous findings. First, BA was used as the outcome variable in this study to evaluate the effect of shortage of or excess sleep duration. Previous studies have demonstrated several findings that are related to sleep duration. Because BA has recently been used as an indicator of health status and aging, we used it to show the effect of shortage of or excess sleep duration on overall health status. Therefore, the results of this study could indicate additional negative problems that are related to sleep. Remedies for this problem should be reviewed to target sleep problems. Second, the data used in this study was nationwide. Therefore, it has strengths related to external validity. The results of this study could be helpful in establishing healthcare alternatives for solving sleep problems in South Korea. Third, our data consisted of both clinical and survey data, allowing for the consideration of several characteristics related to sleep duration. 

However, our study has some limitations related to the nature of the data. First, data that was used in this study was collected with a cross-sectional study design. Therefore, there are some problems related to the causal relationship between sleep duration and difference with BA; the excessive sleep could be caused by worsening health status. The results should be interpreted with caution. Second, in this study, based on a self-reporting survey, sleep duration was measured as response for “How many hours do you usually sleep?” This measurement can cause inaccuracies, including recall bias, because it is not objective sleep duration measurements, such as using actigraphy. However, due to the national research, there were limitations in making elaborate research, and the overall results of sleep duration were similar to previous findings. Third, BA was used as an outcome variable in this study. Several types of BA can be used to measure different targeted diseases. We used the BA related to metabolism to investigate worsening effects that are caused by shortage of or excess sleep duration. Thus, the results of this study could be limited in specific health outcomes and might not estimate some symptoms. Fourth, in previous studies, sleep quality was also associated with health outcomes. Due to limited data, this study only focused on sleep duration, not sleep quality. Therefore, the effect of sleep quality was not measured. Finally, the data used in this study was only collected from South Korean. Thus, we could not capture the diversity of ethnic, and our results cannot be generalized in other ethnics.

Despite some limitations in this study, our findings suggest that excess sleep duration has a positive correlation with higher BA, and this association is greater in vulnerable populations, such as those with low income, obesity, or chronic diseases. Therefore, strategies for managing optimal sleep duration in South Korea should be evaluated and compared to those of other OECD countries. To target efficient alternatives, healthcare professionals should focus on economic or clinically vulnerable populations.

## 5. Conclusions

Excess sleep duration is associated with increased BA. In particular, this relationship of worsened BA was greater in patients who are low income, obese, and have chronic diseases. Based on our findings, healthcare professionals should consider the negative effects of excess sleep, not only sleep shortage. To solve such problems, alternatives to promote optimal sleep duration should be reviewed, particularly in vulnerable populations.

## Figures and Tables

**Table 1:** General characteristics of study population.

Variables	N	%
**Sleep duration**		
<6	4652	14.22
6~8	22,602	78.68
>8	2055	7.10
**Sex**		
Male	12,529	49.44
Female	16,780	50.56
**Age (years)**		
<30	3120	17.40
30~39	5209	20.31
40~49	5534	22.41
50~59	6028	20.03
60≤	9418	19.86
**Educational level**		
Under high school graduation	18,158	55.65
Bachelor’s degree	9899	39.83
Master’s degree or above	1252	4.52
**Marital status**		
Married	22,027	70.62
Marriage problems	3599	9.61
Single	3683	19.78
**Economic activity**		
Unemployed	11,644	34.89
Employed	17,665	65.11
**Household income**		
Low	5307	14.41
Mid-low	7524	26.04
Mid-high	8126	29.80
High	8352	29.75
**BMI**		
<23	12,807	44.21
23–25	6984	23.27
>25	9518	32.52
**Aerobic exercise habits**		
Yes	8304	31.40
No	21,005	68.60
**Smoking status**		
Smoker	5722	24.42
Ex-smoker	6000	20.18
Non-smoker	17,587	55.40
**Alcohol intake**		
Less than twice a week	26,273	86.94
More than twice a week	3036	13.06
**Stress awareness**		
Low	21,916	73.32
High	7393	26.68
**Hypertension**		
Diagnosed	6558	16.80
None	22,751	83.20
**Dyslipidemia**		
Diagnosed	3720	9.92
None	25,589	90.08
**Diabetes Mellitus**		
Diagnosed	2447	6.36
None	26,862	93.64
**Survey year**		
2010	4921	13.92
2011	5568	18.01
2012	5162	17.75
2013	4751	17.02
2014	4352	16.24
2015	4555	17.06
**Total**	29,309	100.00

**Table 2:** The averages of biological age (BA) and its differences with chronological age (CA) in study population.

Variables	BA			Difference (BA-CA)		
	Mean	SD	*p*-Value	Mean	SD	*p*-Value
**Sleep duration**						
<6	61.53	21.12	<0.0001	4.29	14.88	<0.0001
6~8	52.94	21.23		3.75	14.14	
>8	54.62	24.74		4.62	14.63	
**Sex**						
Male	54.52	21.32	<0.0001	3.62	14.68	<0.0001
Female	54.35	21.98		4.10	14.00	
**Age (years)**						
~30	24.60	12.59	<0.0001	0.34	11.80	<0.0001
30~39	38.19	14.04		3.24	13.70	
40~49	49.21	15.48		4.86	15.12	
50~59	60.47	15.20		6.08	14.95	<0.0001
60+	72.47	14.50		3.47	14.16	
**Educational level**						
Under high school graduation	62.04	19.51	<0.0001	4.92	14.91	<0.0001
Bachelor’s degree	41.28	19.15		2.12	13.04	
Master’s degree or above	47.87	18.86		3.05	13.23	
**Marital status**						
Married	56.23	19.22	<0.0001	4.24	14.20	0.0159
Marriage problems	69.68	19.00		5.06	15.73	
Single	28.68	16.62		0.69	12.88	
**Economic activity**						
Unemployed	58.16	23.09	<0.0001	3.78	14.01	0.5913
Employed	51.96	20.37		3.97	14.48	
**Household income**						
Low	67.76	19.80	<0.0001	4.32	15.00	0.0051
Mid-low	55.26	22.14		4.47	15.07	
Mid-high	50.28	20.48		3.91	13.75	
High	49.23	19.93		3.09	13.60	
**BMI**						
<23	44.65	20.08	<0.0001	−3.28	11.04	<0.0001
23–25	57.30	19.49		4.46	12.30	
>25	65.45	19.32		13.14	14.11	
**Aerobic exercise habits**						
Yes	51.39	21.47	<0.0001	3.06	14.06	<0.0001
No	55.62	21.68		4.22	14.37	
**Smoking status**						
Smoker	51.85	21.38	<0.0001	5.21	15.85	<0.0001
Ex-smoker	58.51	20.23		3.51	13.97	
Non-smoker	53.86	22.10		3.60	13.84	
**Alcohol intake**						
Less than twice a week	54.61	21.59	<0.0001	3.41	13.82	<0.0001
More than twice a week	52.82	22.61		8.05	17.33	
**Stress awareness**						
Low	55.24	21.49	0.0827	3.63	13.97	0.0044
High	52.00	22.15		4.67	15.20	
**Hypertension**						
Diagnosed	72.75	15.80	<0.0001	8.47	14.82	<0.0001
None	49.14	20.25		2.58	13.86	
**Dyslipidemia**						
Diagnosed	69.89	17.03	0.073	8.80	15.93	<0.0001
None	52.17	21.39		3.18	13.90	
**Diabetes Mellitus**						
Diagnosed	80.33	18.51	<0.0001	16.45	18.62	<0.0001
None	52.06	20.40		2.75	13.26	
**Survey year**						
2010	57.28	20.04	<0.0001	5.01	14.01	<0.0001
2011	54.01	22.27		3.47	14.59	
2012	54.42	21.90		3.61	14.27	
2013	52.16	21.74		3.57	14.22	
2014	53.15	21.45		3.23	14.05	
2015	55.41	22.32		4.50	14.50	
**Total**	49.75	0.22		4.00	0.12	

**Table 3:** The results of multiple linear regression analysis for the association between sleep duration and differences in biological age (BA) with chronological age (CA).

Variables	Differences		
	β	SE	*p*-Value
**Sleep duration**			
<6	−0.376	0.239	0.1158
6~8	Ref	-	-
>8	1.308	0.343	0.0001
**Sex**			
Male	−3.521	0.234	<0.0001
Female	Ref	-	-
**Age (years)**			
~30	4.039	0.453	<0.0001
30~39	4.989	0.324	<0.0001
40~49	5.462	0.312	<0.0001
50~59	4.522	0.272	<0.0001
60+	Ref	-	-
**Educational level**			
Under high school graduation	0.440	0.384	0.2528
Bachelor’s degree	−0.393	0.370	0.2881
Master’s degree or above	Ref	-	-
**Marital status**			
Married	Ref	-	-
Marriage problems	−0.137	0.349	0.6958
Single	−0.383	0.373	0.3054
**Economic activity**			
Unemployed	Ref	-	-
Employed	−0.319	0.179	0.0745
**Household income**			
Low	0.363	0.319	0.2555
Mid-low	0.528	0.257	0.0401
Mid-high	0.421	0.223	0.0600
High	Ref	-	-
**BMI**			
<23	Ref	-	-
23–25	7.794	0.215	<0.0001
>25	16.539	0.217	<0.0001
**Aerobic exercise habits**			
Yes	−1.363	0.194	<0.0001
No	Ref	-	-
**Smoking status**			
Smoker	Ref	-	-
Ex-smoker	−1.620	0.276	<0.0001
Non-smoker	−2.122	0.280	<0.0001
**Alcohol intake**			
Less than twice a week	Ref	-	-
More than twice a week	3.257	0.403	<0.0001
**Stress awareness**			
Low	Ref	-	-
High	0.738	0.213	0.0006
**Hypertension**			
Diagnosed	2.470	0.274	<0.0001
None	Ref	-	-
**Dyslipidemia**			
Diagnosed	1.464	0.330	<0.0001
None	Ref	-	-
**Diabetes Mellitus**			
Diagnosed	12.879	0.476	<0.0001
None	Ref	-	-
**Survey year**			
2010	−0.077	0.321	0.8091
2011	−1.208	0.314	00.0001
2012	−0.962	0.347	0.0057
2013	−1.281	0.318	<0.0001
2014	−0.438	0.306	0.1527
2015	Ref	-	-
